# Lonicerae Japonicae Flos Attenuates Neutrophilic Inflammation by Inhibiting Oxidative Stress

**DOI:** 10.3390/antiox11091781

**Published:** 2022-09-09

**Authors:** Kuei-Hung Lai, Yu-Li Chen, Mei-Fang Lin, Mohamed El-Shazly, Yu-Chia Chang, Po-Jen Chen, Chun-Han Su, Yen-Chun Chiu, Amina M. Illias, Chih-Chuan Chen, Lo-Yun Chen, Tsong-Long Hwang

**Affiliations:** 1 PhD Program in Clinical Drug Development of Herbal Medicine, College of Pharmacy, Taipei Medical University, Taipei 11031, Taiwan; 2 Graduate Institute of Pharmacognosy, College of Pharmacy, Taipei Medical University, Taipei 11031, Taiwan; 3 Traditional Herbal Medicine Research Center, Taipei Medical University Hospital, Taipei 11031, Taiwan; 4 Research Center for Chinese Herbal Medicine, College of Human Ecology, Chang Gung University of Science and Technology, Taoyuan 33303, Taiwan; 5 Graduate Institute of Health Industry Technology, College of Human Ecology, Chang Gung University of Science and Technology, Taoyuan 33303, Taiwan; 6 Graduate Institute of Natural Products, College of Medicine, Chang Gung University, Taoyuan 33302, Taiwan; 7 Department of Pharmacognosy, Faculty of Pharmacy, Ain-Shams University, Organization of African Unity Street, Abassia, Cairo 11566, Egypt; 8 Department of Pharmaceutical Biology, German University in Cairo, Cairo 11432, Egypt; 9 Department of Medical Research, E-Da Hospital, Kaohsiung 824005, Taiwan; 10 Department of Food Science, College of Human Ecology, Fu Jen Catholic University, New Taipei City 24205, Taiwan; 11 Department of Anesthesiology, Chang Gung Memorial Hospital, Taoyuan 33305, Taiwan; 12 Graduate Institute of Clinical Medical Sciences, College of Medicine, Chang Gung University, Taoyuan 33303, Taiwan; 13 Department of Chemical Engineering, Ming Chi University of Technology, New Taipei City 24301, Taiwan

**Keywords:** Lonicerae japonicae flos, human neutrophils, superoxide anion (O_2_^•−^), calcium mobilization, SARS-CoV-2-Spike/ACE2, bioassay-coupled chromatographic profile

## Abstract

Lonicerae japonicae flos (LJ) is an Asian traditional herb that is used as a dietary supplement, tea, and beverage to clear heat and quench thirst. However, no studies investigated its effect on activated human neutrophils, which played a crucial role in the bad prognosis of coronavirus disease of 2019 (COVID-19) patients by aggravating lung inflammation and respiratory failure. Herein, we evaluated the anti-inflammatory effect of LJ ethanol extract (LJEE) on human neutrophils activated by *N*-formyl-methionyl-leucyl-phenylalanine (fMLF). Our experimental results indicated that LJEE suppressed fMLF-activated superoxide anion (O_2_^•−^) generation, the expression of CD11b, and cell adhesion and migration, as well as the formation of neutrophil extracellular traps in human neutrophils. Further in-depth mechanical investigation revealed that pretreatment with LJEE accelerated the Ca^2+^ clearance, but did not affect the phosphorylation of mitogen-activated protein kinases (MAPKs) and protein kinase B (Akt) in activated human neutrophils. In addition, LJEE displayed a dose-dependent reactive oxygen species (ROS) scavenger activity, which assisted its anti-inflammatory activity. From the bioassay-coupled chromatographic profile, chlorogenic acids were found to dominate the anti-inflammatory effects of LJEE. Moreover, LJ water extract (LJWE) demonstrated an interrupting effect on the severe acute respiratory syndrome coronavirus-2 spike protein (SARS-CoV-2-Spike)/angiotensin-converting enzyme 2 (ACE2) binding. In conclusion, the obtained results not only supported the traditional use of LJ for heat-clearance, but also suggested its potential application in daily health care during the COVID-19 pandemic.

## 1. Introduction

Lonicerae japonicae flos (LJ) is the dried flower buds of *Lonicera japonica* Thunb., which is known as honeysuckle in Japan and Jing Yin Hua (Ren Dong) in Chinese. According to “The Statistics from the National Economic Forest Association”, the global market demand for LJ reached more than 20 million kilograms, with an output value up to USD 860 million. LJ was reported in the ancient pharmacopeia “Ben Cao Gang Mu” [Compendium of Materia Medica, published in China, 1596] as a remedy for exo-pathogenic wind-heat, epidemic febrile, and infectious diseases [1]. Based on its effect on heat-clearing and detoxification, it has been used as a dietary supplement, tea, or beverage for millennia [2]. Recent scientific studies addressed its therapeutic and tonic activities by evaluating its anti-inflammatory [3], antioxidant [3], anti-viral [4], anti-cancer [5], immunomodulatory [6], anti-bacterial [7], analgesic [8], anti-allergic [9], and anti-liver fibrotic effects [10]. The in vivo biological effects of LJ against autoimmune diseases were studied by evaluating its activity in a lipopolysaccharide (LPS)-induced septic inflammation mouse model [11], as well as in an LPS-induced chronic obstructive pulmonary disease (COPD)-like animal model [12,13]. Recent scientific studies were also dedicated to the phytochemistry characterization of LJ, revealing that iridoids presented the most abundant chemical diversity, followed by organic acids, flavonoids, and triterpenoid saponins [1,14]. In addition, essential oils were also one of the important components of LJ, mainly found in the aerial parts, flowers, leaves, and vines [1].

When the coronavirus disease of 2019 (COVID-19) pandemic emerged, LJ was promoted as a potential remedy to deal with COVID-19, due to its effect of dispersing wind, discharging heat, and resolving dampness [15]. Its major constituent, chlorogenic acid, was also predicted to be active in targeting severe acute respiratory syndrome coronavirus 2 (SARS-CoV-2), based on molecular modeling and network pharmacology approaches [16,17,18]. LJ was tested against SARS-CoV-2 in vitro [19]. The results suggested its potential application as a supplement to improve the body’s response to COVID-19 infection.

Recent findings indicated that 20% of COVID-19 (coronavirus disease 2019) patients suffered from complications of acute respiratory distress syndrome (ARDS), resulting in a mortality rate of 63% [20]. ARDS is the severe inflammation of alveolar epithelial cells and pulmonary microvascular endothelial cells during serious infections, shock, trauma, and burns, which leads to diffused alveolar damage, hypoxic respiratory failure, or even death for 30–70% of the patients [21]. The activation and recruitment of neutrophils are key events in the inflammatory response of ARDS [22], suggesting the importance of neutrophils as potential targets to reduce ARDS mortality.

The first line of innate immunity defense is neutrophils that eliminate foreign pathogens. During neutrophilic inflammation, the activated neutrophils move to inflamed tissues and participate in the inflammatory responses through multiple pathways, such as degranulation, oxidative burst, and neutrophil extracellular trap formation [23]. Tissue damage can be caused by the over-activation of neutrophils by the excessive release of reactive oxygen species (ROS), proteases, or neutrophil extracellular traps (NETs), resulting in inflammatory disorders, including autoimmune diseases [24], obstructive pulmonary diseases [25], ARDS [26], and skin diseases [27]. In order to tackle exaggerated inflammatory responses, over-activated neutrophils should be targeted. Natural products proved effective in this task, with potent activity and a favorable safety profile. Fortified extracts, dietary supplements, and functional foods were introduced to target neutrophilic inflammation, with impressive results [28].

To clarify the neutrophilic inhibitory activity of LJ and to provide scientific evidence for its medicinal and functional applications targeting neutrophilic inflammation, the anti-inflammatory effect of LJ ethanol extract (LJEE) on *N*-formyl-methionyl-leucyl-phenylalanine (fMLF)-induced activation of human neutrophils was evaluated. Neutrophilic inflammatory responses, including degranulation, respiratory burst, and chemotactic adhesion and migration, were studied, along with their in-depth mechanisms (calcium mobilization) using a spectrofluorometer, flow cytometry, and Western blot analysis. To identify the main active constituents, we developed a bioassay-coupled high-performance liquid chromatography (HPLC) approach to construct the LJEE biochemical profile. The anti-inflammatory constituents of LJEE were identified and were further quantified using a multiple reaction monitoring tandem mass spectrometric approach (MRM-MS/MS). Calcium mobilization modulated the anti-inflammatory activity of LJEE. This study illustrated the biological activity of LJEE in targeting neutrophil-dominant inflammatory diseases. We also investigated the effects of LJ water extract (LJWE) on the homogeneous time-resolved fluorescence (HTRF) SARS-CoV-2 Spike/angiotensin-converting enzyme 2 (ACE2) binding assay to validate the suggested activity against COVID-19.

## 2. Materials and Methods

### 2.1. Chemicals and Reagents

Cytochalasin B (CB), cytochrome *c*, dextran, triton-X-100, DMSO, fMLF, 1,1-diphenyl-2-picryl-hydrazyl (DPPH), xanthine oxidase, and *α*-tocopherol were obtained from MilliporeSigma (St. Louis, MO, USA). Hydroethidine (HE) and Hank’s balanced salts solution (HBSS) were acquired from Thermo Fisher Scientific Inc. (Waltham, MA, USA). Biological Industries (Beth Haemek, Israel) was the source of trypan blue. *N*-Methoxysuccinyl-Ala-Ala-Pro-Val *p*-nitroanilide was obtained from Calbiochem Research Biochemicals (La Jolla, CA, USA). We purchased 2-(4-Iodophenyl)-3-(4-nitrophenyl)-5-(2,4-disulfophenyl)-2H-tetrazolium monosodium salt (WST-1) from Dojindo Molecular Technologies, Inc. (Kumamoto, Japan). Ficoll-paque plus was procured from GE Healthcare Systems (Little Chalfont, Buckinghamshire, UK). The antibodies against protein kinase B (Akt), phospho-Akt (Ser473), phospho-p38, p38 mitogen-activated protein kinase (MAPK), *c*-Jun N-terminal kinase (JNK), phospho-JNK, extracellular signal-regulated kinase (ERK), and phospho-ERK were acquired from Cell Signaling Technology (Beverly, MA, USA). The fluo-3-acetomethoxyester was acquired from Molecular Probes biotechnology company (Eugene, OR, USA). Anti-CD11b antibodies were bought from Abcam (Abcam, Cambridge, MA, USA).

### 2.2. Preparation of Human Neutrophils

Human donors (20–35 years old) provided blood by venipuncture. The protocol was approved and supervised by the Institutional Review Board (IRB) at Chang Gung Memorial Hospital. The purification of neutrophils was achieved according to a previously reported method [29]. The protocol involved dextran sedimentation, hypotonic lysis, and Ficoll Hypaque gradient of erythrocytes. After the isolation of human neutrophils, they were suspended in a 50 mL centrifuge tube in a calcium (Ca^2+^)-free and magnesium (Mg^2+^)-free HBSS buffer (KH_2_PO_4_: 60 mg/L; KCl: 400 mg/L; NaCl: 8000 mg/L; NaHCO_3_: 350 mg/L; dextrose: 1000 mg/L). The pH was adjusted to 7.4, and the trypan blue exclusion method was used to examine the neutrophils (>98% viable cells). The assessment of the neutrophils was achieved in HBSS (1 mM CaCl_2_ contained) at 37 °C.

### 2.3. Sample Extraction and Preparation

The granules of Lonicerae japonicae flos aqueous extract (Product No. E1228, Batch No. A0845703) were obtained from ChuangSongZong Pharmaceutical Co., Ltd. (Kaohsiung city, Taiwan). For the sample preparation, 10 g of LJ granules were dissolved in 95% ethanol (*w*/*v* = 1/10), distilled water (*w*/*v* = 1/10), and methanol (*w*/*v* = 1/10), using an ultrasonic tank for 15 min, which afforded LJEE (8.83% *w*/*w*), LJWE (14.46% *w*/*w*), and LJME (10.22% *w*/*w*) extracts, respectively. All extracts were evaporated under reduced pressure at 40 °C. Three extracts were dissolved in dimethyl sulfoxide (DMSO) (LJEE and LJME) or distilled water (LJWE) and were sterilized using a 0.45 μm micron filter.

### 2.4. Fraction Preparation for Bioassay-Coupled High-Performance Liquid Chromatography (HPLC) Profile

A Shimazu preparative HPLC system (Shimazu, Kyoto, Japan) was used for profiling the LJEE extract. A COSMOSIL 5C18-MS-II Waters (20 × 250 mm, C18) column (Nacalai Tesque, Kyoto, Japan) was used for the liquid chromatography experiments. Methanol (MeOH, A) and water (W, containing 0.1% formic acid) were mixed and were used as the mobile phase as follows: 0–30 min, 10–60% A; 30–60 min, 60–80% A; 60–70 min, 80–100% A; 70–85 min, 100% A. The temperature of the column was maintained at 35 °C, the mobile phase flow rate was fixed at 10 mL/min, and compounds were detected at wavelengths from 190 to 500 nm. The fractionation sample was prepared using a 1 mg drug sample dissolved in 10 μL of methanol that was filtered using a 0.45 μm membrane filter, and then was loaded into the column. The sample injection was applied manually (100 μL volume per injection). Fractions were collected every 5 min of the retention time window. The collected fractions were subjected to a bioassay.

### 2.5. Ultra-Performance Liquid Chromatography-Tandem Mass Spectrometry (UPLC-MS/MS) Condition

A qualitative and quantitative analysis of LJEE extract was performed using a Shimazu NexeraX2 UPLC (Shimazu, Kyoto, Japan). A Thermo Hypersil GOLD C18 (1.9 µm, 2.1 mm × 100 mm) column (Waltham, MA, USA) was used for the liquid chromatography experiments. MeOH (A) and water (W, containing 0.1% formic acid) were mixed and were used as follows: 0–5 min, 10% A; 5–20 min, 10–40% A; 20–30 min, 10% A. The temperature of the column was maintained at 35 °C, and the flow rate was fixed at 0.5 mL/min. For the sample preparation, 1 mg LJEE extract was dissolved in methanol (1 mL) and was filtered using a 0.45 μm filtering membrane, and then loaded into the column. Then, the sample injection (1 μL volume per) was applied automatically.

The mass spectrometer Shimazu LCMS-8045 was used for product ion scanning (positive or negative mode) and multiple reaction monitoring (MRM). At 100 msec, the dwell time was set, and then the collision energy was automatically optimized for each compound individually. LCMS LabSolutions software (Version 5.93, Shimazu, Kyoto, Japan) was used to process the obtained MS.

### 2.6. Determination of Superoxide Anion (O_2_^•−^) Generation

The superoxidase dismutase (SOD) inhibitable reduction of ferricytochrome *c* was used to determine O_2_^•−^ generation [30]. Neutrophils (6 × 10^5^ cells/mL) were supplemented with ferricytochrome *c* (0.6 mg/mL), were equilibrated at 37 °C, and were incubated for 5 min before treatment with the LJEE extract (3, 10, and 30 μg/mL), pure compounds (3, 10, and 20 μM), or DMSO (0.1%, as the control). To magnify the reaction, 1 μg/mL cytochalasin B (CB) was added and was left for 3 min before the activation (with 0.1 μM fMLF). Any change in the absorbance associated with the reduction of ferricytochrome *c* was monitored continuously at 550 nm (in 4.5 mL cuvette) using a spectrophotometer (U-3010, Hitachi, Tokyo, Japan).

### 2.7. Determination of Elastase Release

An elastase release assay was used for the determination of the degranulation of azurophilic granules. The used substrate of elastase was MeO-Suc-Ala-Ala-Pro-Val-*p*-nitroanilide [31]. Neutrophils (6 × 10^5^ cells/mL) were supplemented with MeO-Suc-Ala-Ala-Pro-Val-*p*-nitroanilide (100 μM), were equilibrated at 37 °C, and were incubated for 5 min before treatment with LJEE extract (3, 10, and 30 μg/mL) and pure compounds (3, 10, and 20 μM). To magnify the reaction, CB (0.5 g/mL) was added. To induce cell activation, fMLF (0.1 μM) was used. To determine the elastase release, the variations in the absorbance at 405 nm (in 4.5 mL cuvette) were monitored continuously.

### 2.8. Determination of Lactate Dehydrogenase (LDH) Release

A cell-free medium was used to assess the cytotoxicity against neutrophils as the ratio of the LDH released in total [28]. At 37 °C, the neutrophils (6 × 10^5^ cells/mL) were equilibrated and were incubated for 5 min before treatment with the LJEE extract (1, 3, 10, and 30 μg/mL). The treatment with the extract lasted 15 min. The cells were then lysed using Triton X-100 (0.1%) at 25 °C for 30 min. The LDH reagent was added, and any changes in the absorbance at 492 nm (in 4.5 mL cuvette) were continuously monitored.

### 2.9. Determination of Superoxide Anion (O_2_^•−^) Scavenging Activity

A cell-free system was used to assess the O_2_^•−^ scavenging ability of LJEE based on examining the WST-1 reduction [28] in xanthine/xanthine oxidase. To the assay buffer [50 mM Tris (pH 7.4)], 0.02 U/mL xanthine oxidase and 0.3 mM WST-1 were added, followed by the addition of xanthine (0.1 mM) at 30 °C for 10 min. LJEE extract (1, 3, 10, and 30 μg/mL) or SOD (20 U/mL) was added to react with xanthine oxidase for 3 min. A spectrophotometer (U-3010, Hitachi, Tokyo, Japan) was used to measure the correlative absorbance of the O_2_^•−^-induced WST-1 reduction. The measurement was performed at 450 nm (in 4.5 mL cuvette).

### 2.10. Assessment of 1,1-Diphenyl-2-Picrylhydrazyl (DPPH) Radical Scavenging Activity

In 100 μM of 99% ethanol, DPPH was dissolved and was incubated with vitamin E (α-tocopherol, the positive control; 1, 3, 5, and 10 μg/mL) or LJEE extract (1, 3, 10, and 30 μg/mL) at 25 °C for 15 min [30]. A spectrophotometer (U-3010, Hitachi, Tokyo, Japan), was used to measure the absorbance variations at 517 nm (in 4.5 mL cuvette).

### 2.11. CD11b Expression

At 37 °C, neutrophils (2.5 × 10^6^ cells/mL) were equilibrated for 5 min before the treatment with LJEE extract (3, 10, and 30 μg/mL) [30]. To activate the neutrophils, fMLF (0.1 μM) and CB (0.5 μg/mL) were added sequentially. Ice was used to quench the reaction for 5 min. Then, the cell pellets were obtained after centrifugation (200× *g*) at 4 °C and were resuspended in HBSS containing 0.5% bovine serum albumin (BSA). Fluorescein isothiocyanate (FITC)-conjugated anti-CD11b antibodies (1 μg) were added and were reacted under a light-proof environment at 4 °C for 90 min. Flow cytometry was used to detect the fluorescence intensity.

### 2.12. Determination of Neutrophils Adhesion

Hoechst 33342 (1 ng/mL) was used to label neutrophils (5 × 10^6^ cells/mL), and the temperature was maintained at 37 °C. The LJEE extract (30 μg/mL) was added to the labeled neutrophils for another 5 min [28]. At 37 °C, fMLF (0.1 μM)/CB (1 μg/mL) was used to activate the neutrophils. They were then co-cultured with bEnd.3 endothelial cells (ECs) on a 12-well plate for 30 min. To fix the cells, 4% paraformaldehyde was added. The counts of the neutrophils adhering to bEnd.3 cells were counted using a fluorescent microscope (Olympus Corporation, Center Valley, PA, USA).

### 2.13. Neutrophils Chemotactic Migration Assay

At 37 °C, the 5 × 10^6^ cells/mL of incubated neutrophils were treated with LJEE extract (30 μg/mL) or DMSO for 5 min [28]. Then, the suspension of neutrophils was added to a Millicell Culture Plate Insert (pore size 3 μm) (Millipore Darmstadt, Germany). Then, the inserts were put into the dishes (which contained a chemokine solution). Ethylenediaminetetraacetic acid (EDTA) was added after 90 min. The counts of the migrated neutrophils were counted using a fluorescent microscope (Olympus Corporation, Center Valley, PA, USA).

### 2.14. Analysis of Neutrophil Extracellular Trap (NET) Formation

DMSO or LJEE (30 μg/mL) was added to neutrophils (5 × 10^5^ cells/mL) (for 10 min), and the neutrophils were then activated by phorbol 12-myristate 13-acetate (PMA, 10 nM) (for 3 h). An amount of 2.5 μM of SYTOX Green nucleic acid stain was added (for 15 min). At 485–535 nm, the fluorescence was detected (in 4.5 mL cuvette) [29].

### 2.15. Measurement of [Ca^2+^]_i_

At 37 °C, the neutrophils (6 × 10^6^ cells/mL) and fluo-3/AM (2 μM) were incubated for 30 min [28]. The cells were centrifuged (200 *g*) for 8 min at 4 °C. The pellets were acquired to resuspend in HBSS. At 37 °C, the solution of HBSS suspension was then incubated (for 3 min), followed by treatment with LJEE extract (10 and 30 μg/mL). The fMLF (0.1 μM) was added for neutrophil activation. Triton X-100 and ethylene glycol-bis(2-aminoethylether)-*N*,*N*,*N′*,*N′*-tetraacetic acid (EGTA) were added in order to reach the maximum (*F*_max_) and minimum (*F*_min_) fluorescence values, respectively. A spectrofluorometer was used to detect the fluorescence intensity variations at 488 and 520 nm (in 4.5 mL cuvette).

### 2.16. Western Blotting

At 37 °C, the human neutrophils were pretreated with LJEE for 5 min. The cells were activated by fMLF (0.1 μM)/CB (1 μg/mL) [28]. At 100 °C, a sample buffer (62.5 mM pH 6.8 Tris-HCl, 4% sodium dodecyl sulfate, 5% β-mercaptoethanol, 0.0125% bromophenol blue, 8.75% glycerol, 1% protease inhibitor cocktail, and 1% phosphatase inhibitor cocktail) was used to quench the reaction for 15 min. Sodium dodecyl sulfate-polyacrylamide gel electrophoresis was used to separate the cell lysates. Western blotting was used with the relevant primary antibodies (MAPKs and Akt) and with horseradish peroxidase-conjugated secondary antibodies at room temperature for 1 h. The protein levels were then detected by an enhanced chemiluminescence system and a densitometer (UVP, Upland, CA, USA).

### 2.17. The Homogeneous Time-Resolved Fluorescence (HTRF) Severe Acute Respiratory Syndrome Coronavirus 2 Spike Protein (SARS-CoV-2 spike)/Angiotensin-Converting Enzyme 2 (ACE2) Binding Assay

LJ extracts (LJWE: 3, 10, and 30 μg/mL; LJEE: 30 μg/mL; LJME: 30 μg/mL) were mixed with the Tag1-SARS-CoV-2 spike protein (5 nM) and Tag2-ACE2 protein (75 nM). The mixture was incubated for 15 min at room temperature. Then, pre-mixed anti-Tag1-Eu^3+^ (an HTRF donor) and anti-Tag2-d2 (an HTRF acceptor) were added to detect the close binding of SARS-CoV-2 spike/ACE2. After 3 h of incubation in sealed plates, the excitation of the antibody triggered fluorescent resonance energy transfer (FRET) towards the acceptor antibody, which was measured at 665 nm (in 4.5 mL cuvette).

### 2.18. Statistics

**Figure 1:**
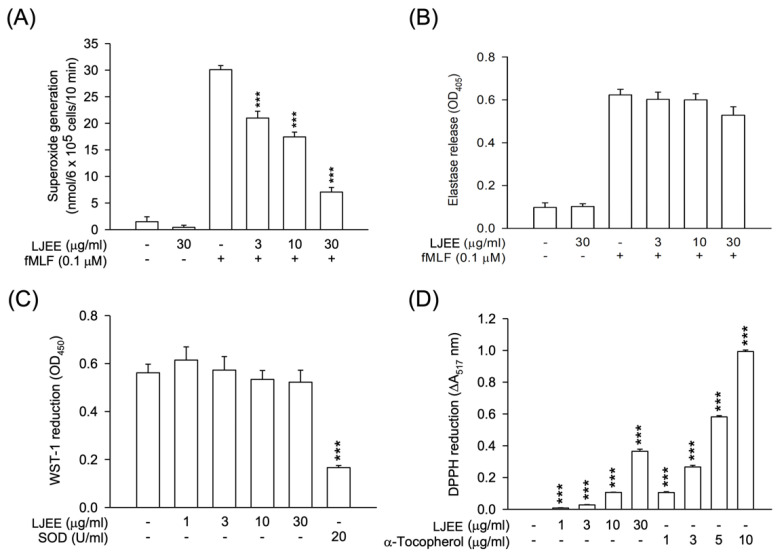
Superoxide anion generation was inhibited by Lonicerae japonicae flos ethanol extract (LJEE), but not elastase release. It exhibited antioxidant activity in *N*-formyl-methionyl-leucyl-phenylalanine (fMLF)-activated human neutrophils. (**A**) Ferricytochrome *c* reduction was used to detect the superoxide anion inhibition by LJEE (3–30 μg/mL). (**B**) The release of elastase was detected spectrophotometrically at 405 nm. A cell-free system was used to assess the antioxidant activity. (**C**) Superoxide-scavenging effect of LJEE (3–30 μg/mL) was detected by the reduction of 2-(4-Iodophenyl)-3-(4-nitrophenyl)-5-(2,4-disulfophenyl)-2H-ter-trazolium monosodium salt (WST-1). (**D**) ROS-scavenging effect of LJEE (3–30 μg/mL) was assessed by the reduction of DPPH (ΔA_517_ nm). All data are presented as the mean ± S.E.M. (*n* = 5–7). *** *p* < 0.001, compared with the DMSO + fMLF (for A and B) or the DMSO (for C and D) alone group.

**Figure 2:**
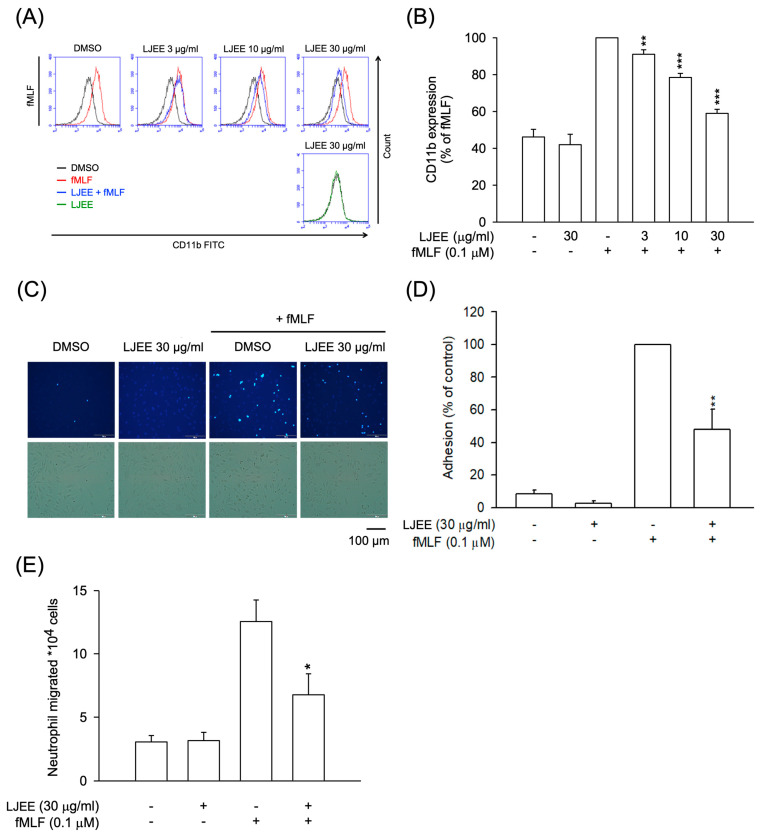
LJEE inhibited CD11b expression, as well as chemotactic migration and adhesion in fMLF-induced human neutrophils. (**A**) A flow cytometry, using Fluorescein isothiocyanate (FITC)-labeled anti-CD11b antibodies, was used to detect the level of CD11b on the cell surface. Mean fluorescence intensities are shown in (**B**). Fluorescent microscopy was used to assess the adherent neutrophils. (**C**) Representative fluorescence (upper panel) and bright (lower panel) field microscopic images. (**D**) Adherent neutrophils were counted and quantified. (**E**) The migrated neutrophils in response to fMLF were determined by cell counter. All the above data are shown as the mean ± S.E.M. (*n* = 5–6). * *p* < 0.05, ** *p* < 0.01, and *** *p* < 0.001, compared with the DMSO + fMLF group.

**Figure 3:**
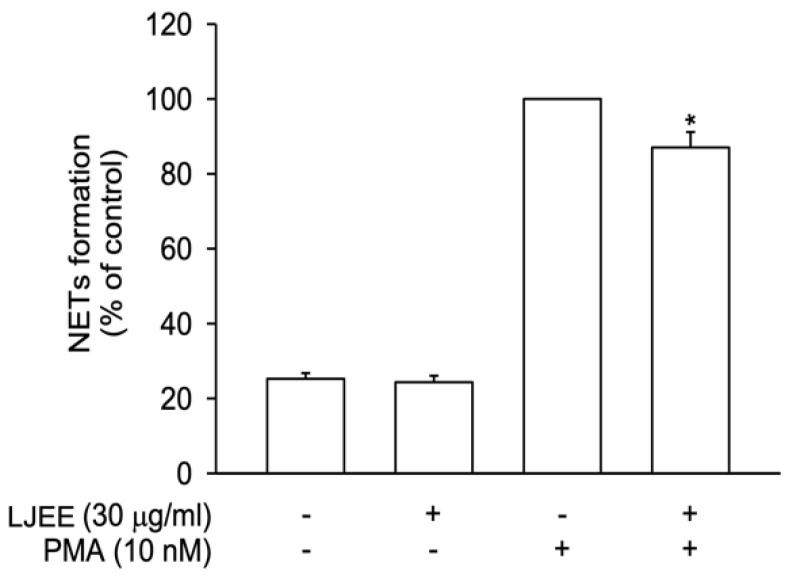
LJEE (30 μg/mL) inhibited neutrophil extracellular traps (NETs) in phorbol 12-myristate 13-acetate (PMA)-stimulated human neutrophils. All data are expressed as the mean ± S.E.M. (*n* = 6). * *p* < 0.05, as compared with the control group.

**Figure 4:**
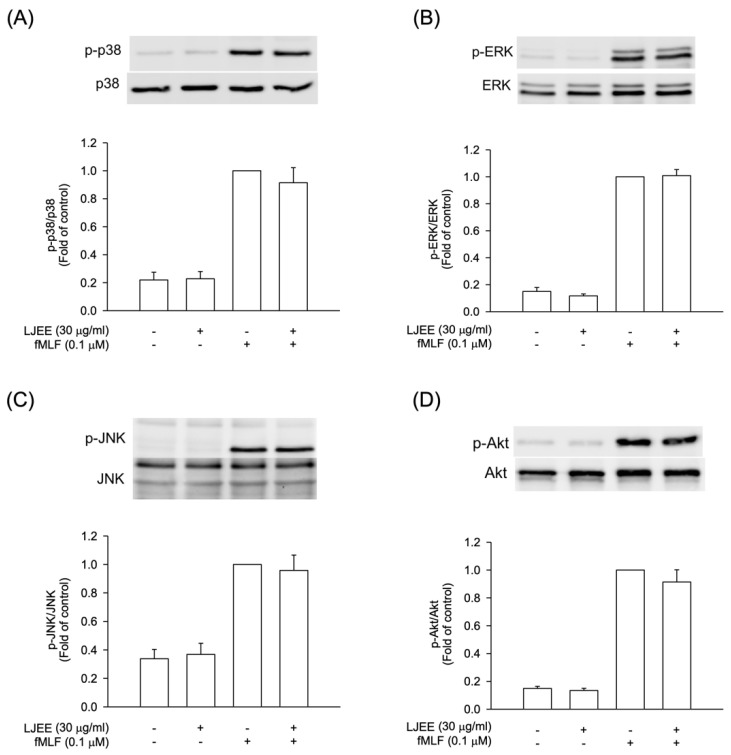
LJEE did not affect the phosphorylation of mitogen-activated protein kinases (MAPKs) and protein kinase B (Akt) in activated human neutrophils. Phosphorylation of (**A**) p38, (**B**) extracellular signal-regulated kinase (ERK), (**C**) *c*-Jun *N*-terminal kinase (JNK), and (**D**) Akt were analyzed by immunoblot, using antibody against the phosphorylated and total protein. All data are shown as the mean ± S.E.M. (*n* = 6 or 7).

**Figure 5:**
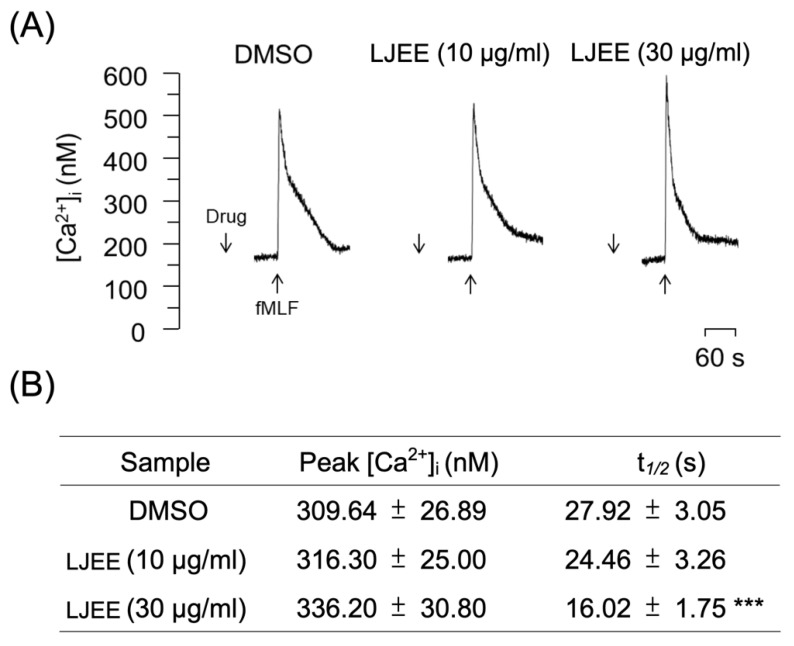
LJEE inhibited the [Ca^2+^]_i_ mobilization in fMLF-induced human neutrophils. (**A**) Representative patterns and (**B**) the [Ca^2+^]_i_ peak and t_1/2_ are shown. Data are expressed as mean ± S.E.M. (*n* = 7). *** *p* < 0.001, compared with the DMSO + fMLF group.

**Figure 6:**
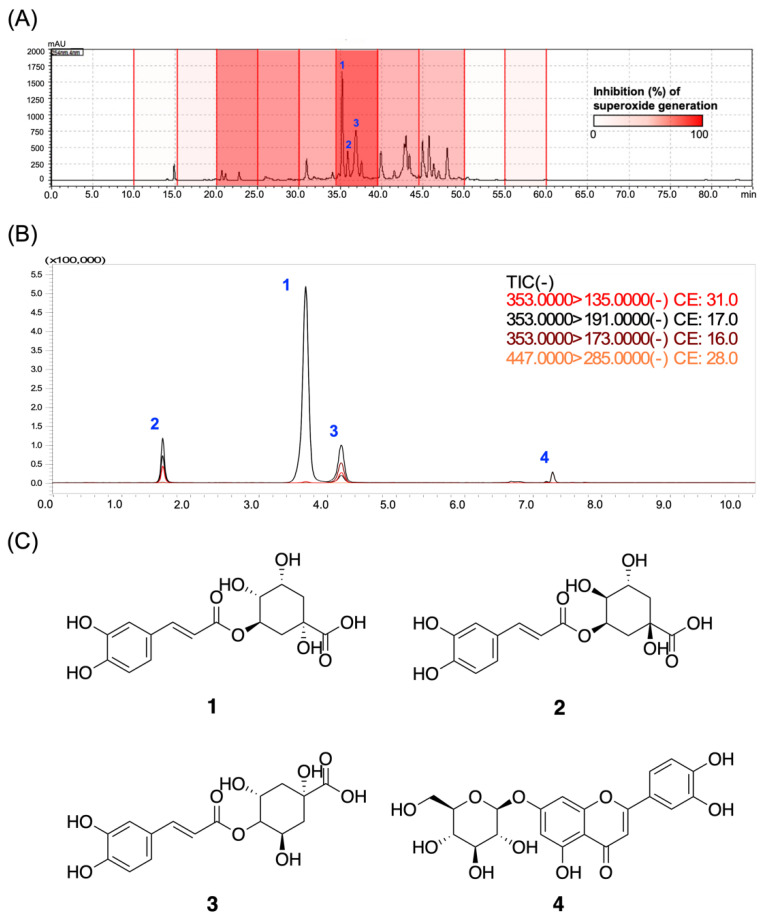
The biological evaluation of the bioactive compounds from LJEE. (**A**) The bioassay-coupled high-performance liquid chromatography (HPLC) profile was performed using the retention time-based fractionation approach, together with the assessments of superoxide anion inhibitions (10 μg/mL). (**B**) The total ion current (TIC) and product ions chromatogram of the LJEE compounds from multiple reaction monitoring (MRM) experiments. (**C**) The chemical structures of the identified chlorogenic acid (**1**), neochlorogenic acid (**2**), cryptochlorogenic acid (**3**), and cynaroside (**4**) from LJEE.

For Figure 1, Figure 2B, Figure 3B and Figure 6, one-way ANOVA and Dunnett’s multiple comparison tests were employed. For Figure 2D–E, Figure 4 and Figure 5, the Tukey test was used. GraphPad Prism software (GraphPad Software version 9, San Diego, CA, USA) was used for all statistical calculations. *p* values < 0.05 were considered to show statistically significant effects.

## 3. Results and Discussions

### 3.1. Lonicerae Japonicae Flos Ehanol Extract (LJEE) Inhibited the Superoxide Anion (O_2_^•−^) Generation but Not Elastase Release in N-Formyl-Methionyl-Leucyl-Phenylalanine (fMLF)-Activated Human Neutrophils

Pathogen-associated molecular patterns (PAMPs) such as fMLF can activate neutrophils, resulting in a series of inflammatory responses, including degranulation (elastase release), respiratory burst (O_2_^•−^ generation), chemotactic adhesion, and migration [32]. To evaluate the anti-inflammatory activity of LJEE extract, we first assessed its effect on respiratory burst and degranulation. Only O_2_^•−^ generation, but not elastase release, was suppressed by the treatments with LJEE (3, 10, and 30 μg/mL) in fMLF-induced human neutrophils (IC_50_ value of 10.16 ± 1.27 μg/mL) (Figure 1A,B). LJEE extract did not show any cytotoxicity on human neutrophils, as demonstrated by the LDH release assay (data not shown). These findings suggested that the therapeutic potential of LJEE extract on neutrophilic inflammation is not due to the cytotoxic effect.

Respiratory burst plays a predominant role in defending foreign pathogens in activated neutrophils. The attenuation of human neutrophils oxidative stress-dependent inflammatory responses is considered a sign of the suppression of respiratory burst [33]. We used the cell-free xanthine/xanthine oxidase system and DPPH assay to evaluate the scavenging effect of LJEE extract against O_2_^•−^ (a reactive oxygen species) or other free radicals of reactive nitrogen species. The treatment with LJEE did not scavenge O_2_^•−^, but it captured reactive nitrogen species (Figure 1C,D). The LJEE extract dose-dependently scavenged free radicals in the DPPH assay. We found that the LJEE-scavenging property in activated human neutrophils was mediated through intracellular signaling modulation. The antioxidant property of LJEE on reactive nitrogen species may assist its anti-inflammatory activity.

In previous reports, the antioxidant activity of LJ was documented [34], and its free radical-scavenging activity was attributed to chlorogenic acid, a major constituent of LJEE [35]. The potent antioxidant effect suggested the potential therapeutic application of LJEE against oxidative stress-dependent inflammatory diseases. We found that LJEE’s biological effect supports its use as an anti-inflammatory remedy and daily health supplement for well-being.

### 3.2. LJEE Ameliorated the fMLF-Induced Neutrophilic Adhesion, Migration, and CD11b Expression

PAMPs stimulate neutrophils, promoting the expression of CD11b/CD18 on the cell membrane. This effect results in neutrophil adhesion to ECs and, subsequently, chemotactic migration [36]. As shown in Figure 2A,B, the fMLF-activated CD11b expression was attenuated by treatment with LJEE extract (3, 10, and 30 μg/mL). Then, the fMLF-activated neutrophilic adhesion (Figure 2C,D) and migration (Figure 2E) were suppressed by LJEE extract (30 μg/mL). These results, taken together, showed that LJEE extract suppressed neutrophil inflammation by inhibiting respiratory burst, chemotactic adhesion, and migration.

### 3.3. LJEE Suppressed NET Formation

NET formation participates in neutrophilic inflammatory disorders [29]. In our current study, NETs were released after stimulation with PMA (10 nM) for 3 h. In Figure 3, LJEE treatment reduced extracellular DNA structure formation, suggesting that the neutrophilic inhibitory effect of LJEE was achieved through the inhibition of NET formation.

### 3.4. The Phosphorylation of Mitogen-Activated Protein Kinases (MAPKs) and Protein Kinase B (Akt) Signaling in Activated Human Neutrophils Were Not Affected by LJEE Treatment

The intracellular signal pathways, such as MAPKs and Akt, were found to play critical roles during neutrophilic inflammation, involved in degranulation, oxidative burst, chemotaxis, and NET formation [37,38]. In order to evaluate the pharmacological effects of LJEE, the activation of Akt and MAPKs were assessed using the Western blotting approach. The pretreatment with LJEE (30 μg/mL) did not affect the fMLF-induced MAPKs and Akt phosphorylations in human neutrophils (Figure 4). Based on these findings, we suggested that the anti-inflammatory effects of LJEE might proceed through mechanisms other than MAPK and Akt signaling pathways.

### 3.5. LJEE Decreased the fMLF-Induced Neutrophilic Intracellular Ca^2+^ Mobilization

In neutrophils, Ca^2+^ is a major secondary messenger that participates in respiratory bursts, degranulation, and cytoskeleton rearrangements. The ligation of fMLF to formyl peptide receptor 1 (FPR1) induces PLC-associated phosphatidylinositol 4,5-bisphosphate (PIP_2_)-inositol triphosphate (IP_3_) hydrolysis. After hydrolysis, it attaches to the IP_3_ receptors on the endoplasmic reticulum (ER), where this binding initiates calcium mobilization from the ER to the cytoplasm [39]. Under physiological conditions, the activation of fMLF leads to a transient elevation in the intracellular calcium levels [Ca^2+^]i up to a maximum level, which withdraws [Ca^2+^]i to equilibrium in the aftermath within a short time (one minute). Our current results revealed that the pretreatment with LJEE extract (3 and 30 μg/mL) did not affect the level of the [Ca^2+^]_i_ peak (Figure 5A,B), but reduced the time demanded for [Ca^2+^]_i_ to backtrack to half of its peak height (t_1/2_; Figure 5B). It has been documented that the pharmacological inhibition of t_1/2_ of calcium mobilization, but not the [Ca^2+^] peak, still successfully alleviates neutrophil activation, including O_2_^•−^ and ROS generation from respiratory burst [40,41,42]. Moreover, the restoration of Ca^2+^ homeostasis was proved to be essential for the prevention of Ca^2+^ overload and cell hyperactivity following the activation of neutrophils. This could be achieved through the rapid clearance of Ca^2+^ by the unified operation of the plasma membrane Ca^2+^-ATPase and endo-membrane Ca^2+^-ATPase [43]. Thus, we suggested that LJEE extract exhibited neutrophilic inhibitory properties by accelerating the Ca^2+^ clearance, and thus affects O_2_^•−^ generation in a respiratory burst.

Store-operated calcium entry (SOCE) was shown to play a critical role in NADPH oxidase regulation, which might produce a burst of O_2_^•−^, resulting in oxidative stress and the further development of inflammation [44]. α-PKC (α-protein kinase C), an element mediated by SOCE, was found to be a selective element in the positive signaling of fMLF-induced superoxide anion generation without affecting elastase release [45]. Therefore, we suggested that the LJEE-mediated intracellular calcium inhibition might selectively attenuate the generation of superoxide anion, but not the concurrent release of elastase, in human neutrophils (Figure 1A,B and Figure 5).

### 3.6. Chlorogenic Acids Derivatives Dominated the Neutrophilic Inhibition of LJEE in fMLF-Induced Human Neutrophils

We further investigated the activity of phytoconstituents as an important requirement for the registration of functional food and herbal medications by regulatory authorities. We first constructed an anti-inflammatory assay-based high-performance liquid chromatography (HPLC) profile to interpret the relationships between the retention time (t*_R_*)-dependent fractions of LJEE and their activity on superoxide anion generation. As shown in the profile (Figure 6A), the most potent anti-inflammatory fraction (t*_R_*: 34.3–39.5 min) exhibited 86.44% inhibition of O_2_^•−^ generation at 10 μg/mL. The isolation, purification, and structure elucidation led to the identification of three major chlorogenic acid derivatives from LJEE, chlorogenic acid (**1**), neochlorogenic acid (**2**), and cryptochlorogenic acid (**3**) (Figure 6C). Among all the isolates, chlorogenic acid (**1**) is considered the major bioactive constituent, as reported in the “Pharmacopoeia of the People’s Republic of China (Pharmacopoeia Commission of the Ministry of Public Health, People’s Republic of China, 2020)” and “Taiwan Herbal Pharmacopeia (Ministry of Health and Welfare, Taiwan, 2019)”. Since the commercially available cynaroside (**4**) was also reported as a component of Lonicerae japonicae flos according to the Pharmacopoeia of the People’s Republic of China, compounds **1**–**4** were assayed.

The obtained major compounds (**1**–**4**) from the active fraction of LJEE were evaluated for their effects on respiratory burst and degranulation. The chlorogenic derivatives (**1**–**3**) showed identical activities on superoxide anion, with IC_50_ values ranging from 3.76 to 3.97 μM, but neochlorogenic acid (**3**) did not affect the elastase release at 20 μM (Table 1). However, the effect of cynaroside (**4**) on activated neutrophils seemed to be negligible, with 52.63% inhibition at 20 μM. The results of the chlorogenic derivatives (**1**–**3**) showed a similar bioassay profile to LJEE, with the significant suppression of superoxide anion scavenging, but not of elastase release.

The qualitative and quantitative analyzing protocols were established to detect the exact amount of those compounds (**1**–**4**) in LJEE extract. Tandem MS shows the high efficiency of compound characterization and quantitative analysis, in particular, the multiple reaction monitoring (MRM) experiment that is designed to detect the specific MS/MS fragmentations from the precursor compound ions based on a multi-quadrupole MS spectroscopy [46]. We hereby performed an MRM experiment on a triple quadrupole MS spectroscopy in order to come up with a rapid and sensitive method for the qualitative and quantitative quantification of the phytoconstituents of LJEE (Table 2, Figure 6B). The optimal detection of these compounds was selected to be the negative (−) mode, and the individual collision energies were optimized to give rise to the maximum response of their daughter ions. The characteristic product ions of each compound were picked for the MRM settings. The peak areas of ion currents versus five concentrations (0.3125, 0.625, 1.25, 2.5, and 5 ppm) of the standard compounds were used to construct the calibration curves. By the developed quantitative protocols, the four compounds (**1**–**4**) were analyzed to be 1.23%, 3.32%, 0.56%, and 0.89%, respectively. Taking into consideration the IC_50_ value of LJEE (10.16 μg/mL) on superoxide anion inhibition, the total content of the sum of the three major chlorogenic acids, 5.11%, contributed a reasonable inhibitory activity (IC_50_ values: 1.33–1.40 μg/mL), indicating that the anti-inflammatory potential of LJEE on activated neutrophils is mainly attributed to its content of chlorogenic acid derivatives.

Apart from the chlorogenic acid derivatives, flavonoids/flavonoids glycosides from LJ, such as luteolin, ochnaflavon, luteolin-7-*O*-β-D-glucopyranoside, and quercetin 3-*O*-β-D-glucopyranoside, were also reported to exhibit anti-inflammatory and antioxidative properties [47,48,49]. Luteolin effectively attenuated the lipopolysaccharide (LPS)-induced tumor necrosis factor-α, interleukin-6, and inducible nitric oxide production in vitro, as well as protected against LPS-induced lethal toxicity by inhibiting pro-inflammatory molecule expression in vivo [47]. Ochnaflavon was found to significantly decrease cyclooxygenase-2 (COX-2)-dependent prostaglandin D2 (PGD2) generation in mast cells, exhibiting dual COX-2/5-1ipoxygenase inhibitory properties [49]. Both luteolin-7-*O*-β-D-glucopyranoside and quercetin 3-*O*-β-D-glucopyranoside were shown to be potent antioxidants in the DPPH scavenging assay [48]. Moreover, in the septic mouse model, an ethanolic crude extract of LJ, HS-23, was found to alleviate septic injury by inhibiting toll-like receptor 4 signaling, evidenced by the downregulation in protein expressions of myeloid differentiation primary response protein 88, p38 and *c*-Jun *N*-terminal kinase, TIR-domain-containing adapter-inducing interferon-*β*, and interferon regulatory transcription factor 3 [50]. Therefore, it is suggested that the anti-inflammatory and antioxidative activities of these ethanol-soluble flavonoid derivatives and crude extracts may also support the use of LJEE in heat-clearing applications.

### 3.7. Lonicerae Japonicae Flos Water Extract (LJWE) Extracts Interrupted the Binding of SARS-CoV-2 Spike/ACE2

**Figure 7:**
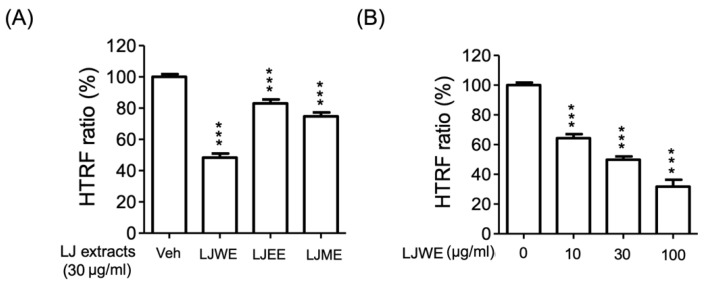
Lonicerae japonicae flos (LJ) extracts interrupted the binding of severe acute respiratory syndrome coronavirus 2 spike protein (SARS-CoV-2 spike)/angiotensin-converting enzyme 2 (ACE2). (**A**) Three LJ extracts (30 μg/mL; LJWE: LJ water extract; LJEE: LJ ethanol extract; LJME: LJ methanol extract); (**B**) LJWE (10–30 μg/mL). *** *p* < 0.001.

The receptor-binding domain (RBD) of SARS-CoV-2 coronavirus spike proteins plays the role of a critical determiner of viral tropism and infectivity [51]. The current study applied an HTRF SARS-CoV-2 spike/ACE2 binding assay kit to determine if the binding levels between the SARS-CoV-2 spike protein and ACE2 protein were affected by LJ extracts. The results indicated that the three LJ extracts (LJWE, LJEE, and LJME), as well as the LJWE (IC_50_: 27.52 ± 1.26 μg/mL), showed a suppressive effect on reducing the SARS-CoV-2 spike/ACE2 binding (Figure 7A,B). This data revealed the interference effect of LJWE on SARS-CoV-2 infection.

## 4. Conclusions

The anti-inflammatory effect and underlying pharmacological mechanisms of Lonicerae japonicae flos (LJ) on activated human neutrophilic inflammation were illustrated for the first time. The chlorogenic acid-enriched LJEE successfully attenuated inflammatory reactions in the activated neutrophils, including superoxide anion generation, release of elastase, CD11b expression, chemotactic migration, cell adhesion, and NET formation. Calcium mobilization played a crucial role in the anti-neutrophilic inflammatory mechanism of action of LJEE. The tandem mass-based qualitative and quantitative analyzing protocols of the phytoconstituents from LJEE were established. Moreover, LJWE was found to interfere with the infecting process of SARS-CoV-2. Since both neutrophilic inflammation and SARS-CoV-2/ACE2 bindings are crucial for COVID-19 infection, the daily use of LJ during the COVID-19 pandemic is suggested.

## Figures and Tables

**Table 1:** The effect of compounds from Lonicerae japonicae flos (LJ) on superoxide anion generation and elastase release in fMLF/CB-induced human neutrophils.

Compounds	Superoxide Anion		Elastase Release	
	IC_50_ (μM) ^a^	Inh (%) ^b^	IC_50_ (μM) ^a^	Inh (%) ^b^
Chlorogenic acid	3.91 ± 0.35	74.63 ± 1.82	8.51 ± 0.47	75.41 ± 3.22
Neochlorogenic acid	3.97 ± 0.58	76.78 ± 3.73	>20	23.82 ± 2.44
Cryptochlorogenic acid	3.76 ± 0.63	75.85 ± 1.85	12.14 ± 3.97	62.05 ± 6.07
Cynaroside	≥20	52.63 ± 5.00	– ^c^	
LY294002 ^d^	1.91 ± 0.56	88.99 ± 1.10	2.94 ± 0.12	83.49 ± 3.47

^a^ Concentration necessary for 50% inhibition (IC_50_); ^b^ percentage of inhibition at 20 μM; ^c^ not detected due to strong interaction with subtracts; ^d^ positive control. Results are presented as mean ± S.E.M. (*n* = 3–7).

**Table 2:** Establishment of the LC-MS/MS analysis methods of the selected compounds from LJEE.

Compounds	Ion Adduct	Precursor/ Product ion (*m/z*) ^a^	CE	Calibration Curve	*R* ^2^	Amount (%)
Chlorogenic acid	[M-H]^−^	353/191	31	Y = 249,834X − 6253	0.9997	3.32
Neochlorogenic acid	[M-H]^−^	353/135	17	Y = 1,113,295X + 3620	0.9997	1.23
Cryptochlorogenic acid	[M-H]^−^	353/173	16	Y = 218,121X + 10,620	0.9993	0.56
Cynaroside	[M-H]^−^	447/285	28	Y = 642,917 X + 167,946	0.9954	0.89

^a^ The product ions were selected for MRM quantitative analysis.

## Data Availability

The data presented in this study are available in the article.
